# Antibiotic-induced microbiome depletion protects against MPTP-induced dopaminergic neurotoxicity in the brain

**DOI:** 10.18632/aging.102221

**Published:** 2019-09-03

**Authors:** Yaoyu Pu, Lijia Chang, Youge Qu, Siming Wang, Kai Zhang, Kenji Hashimoto

**Affiliations:** 1 Division of Clinical Neuroscience, Chiba University Center for Forensic Mental Health, Chiba 260-8670, Japan

**Keywords:** antibiotic, dopamine, gut microbiota, MPTP, neurotoxicity

## Abstract

Although the brain–gut axis appears to play a role in the pathogenesis of Parkinson’s disease, the precise mechanisms underlying the actions of gut microbiota in this disease are unknown. This study was undertaken to investigate whether antibiotic-induced microbiome depletion affects dopaminergic neurotoxicity in the mouse brain after administration of 1-methyl-4-phenyl-1,2,3,6-tetrahydropyridine (MPTP). MPTP significantly decreased dopamine transporter (DAT) immunoreactivity in the striatum and tyrosine hydroxylase (TH) immunoreactivity in the substantia nigra of water-treated mice. However, MPTP did not decrease DAT or TH immunoreactivity in the brains of mice treated with an antibiotic cocktail. Furthermore, antibiotic treatment significantly decreased the diversity and altered the composition of the host gut microbiota at the genus and species levels. Interestingly, MPTP also altered microbiome composition in antibiotic-treated mice. These findings suggest that antibiotic-induced microbiome depletion might protect against MPTP-induced dopaminergic neurotoxicity in the brain via the brain–gut axis.

## INTRODUCTION

Parkinson’s disease (PD) is a common and progressive neurodegenerative disease that predominately affects dopaminergic neurons in the striatum and substantia nigra (SNr) [1, 2]. There is also evidence that loss of dopamine at extrastriatal sites in the basal ganglia, thalamus, or cortex contributes to PD pathology [3]. Although the precise mechanisms underlying PD pathology remain largely unknown, evidence suggests that the brain–gut axis plays a crucial role [4–13]. For example, alterations in bowel function, mainly in the form of constipation, can precede the onset of the prototypical motor symptoms of PD [14].

Over the past two decades, it has become apparent that gut microbiota is a fundamental factor in host physiology and pathology. The brain–gut axis is a complex, multi–organ, bidirectional signaling system involving the gut microbiota and the brain [6, 15–23]. Moreover, although antibiotics are crucial, their overuse plays a role in the pathogenesis of several diseases associated with microbiota impairment [24, 25]. Antibiotic cocktail-induced microbiome depletion has been used to investigate the role of gut microbiota in some pathological conditions [26–35]. In addition, Sampson *et* *al*. [36] reported that gut microbiota are necessary for motor deficits induced by α-synuclein overexpression in mice. Interestingly, antibiotic treatment ameliorated these deficits, while microbial re-colonization promoted PD pathology in mice. Remarkably, colonization of α-synuclein overexpressing mice with microbiota from PD patients enhanced physical impairments compared to microbiota transplants from healthy control subjects. Collectively, these findings suggest that the effects of the brain–gut axis in the pathology of PD are mediated at least in part by the gut microbiota. However, the effects of antibiotic-induced microbiome depletion on dopaminergic neurotoxicity in the brains of PD model mice are unknown.

In this study, we investigated whether antibiotic- induced microbiome depletion affects MPTP (1-methyl-4- phenyl-1,2,3,6-tetrahydropyridine)-induced dopaminergic neurotoxicity, which is widely used as an animal model of PD [37], in the mouse brain.

## RESULTS

### Effects of the antibiotic cocktail on body weight

**Figure 1:**
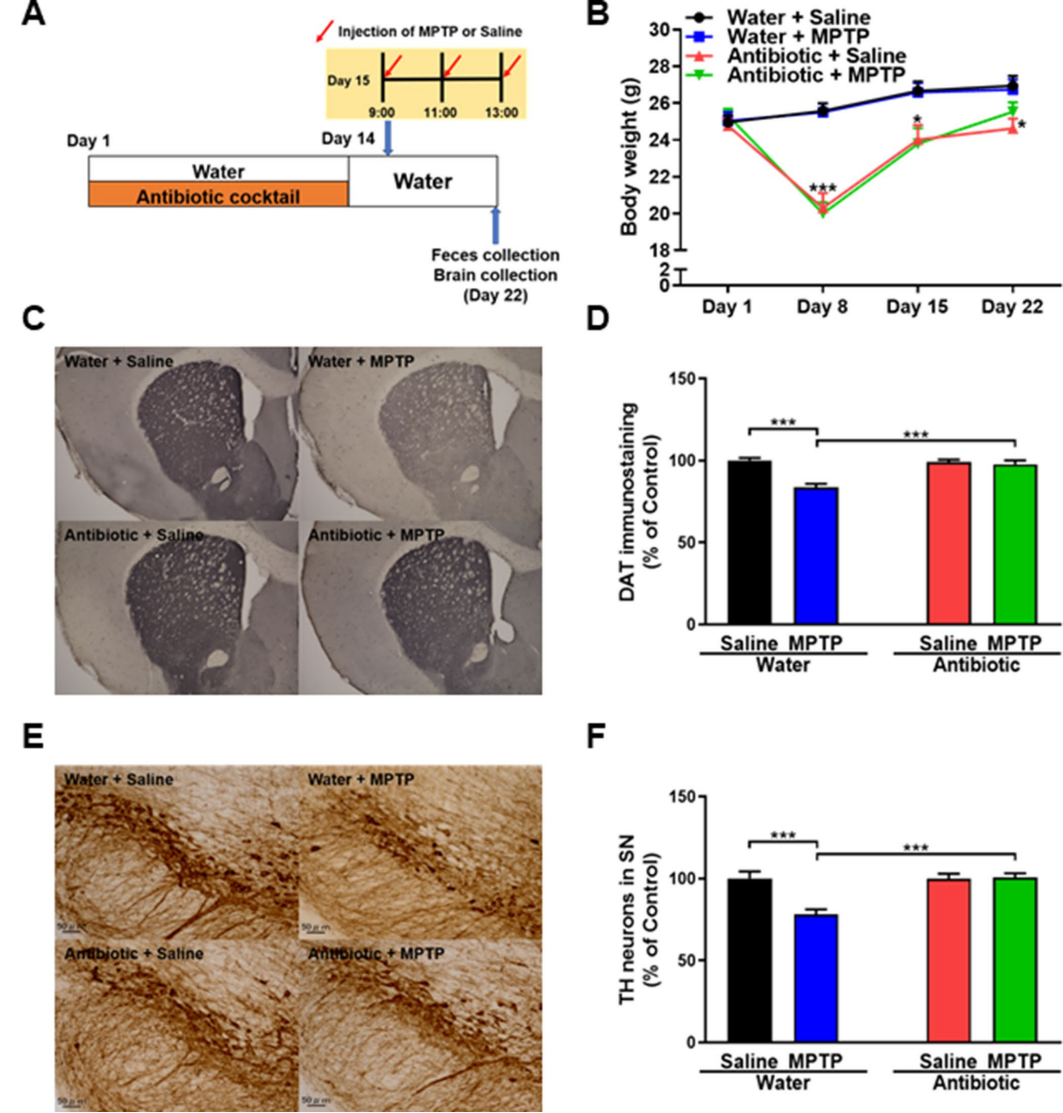
**Effects of antibiotic treatment on gut microbiota diversity.** (**A**) Treatment schedule. Adult mice received drinking water with or without antibiotic cocktail from day 1 to day 14. On day 15, MPTP or saline injections were administered. On day 22, fresh feces were collected. Mice were then perfused for immunohistochemistry. (**B**) Body weights in the different groups (repeated two-way ANOVA, antibiotic: F_1,36_ = 20.549, P < 0.001; MPTP: F_1,36_ = 0.005, P = 0.994; interaction (antibiotic × MPTP): F_1,36_ = 0.045, P = 0.833). (**C**) Representative images of DAT immunohistochemistry in the water + saline, water + MPTP, antibiotic + saline, and antibiotic + MPTP groups. (**D**) Striatal DAT immunoreactivity data. (**E**) Representative images of TH immunohistochemistry in the water + saline, water + MPTP, antibiotic + saline, and antibiotic + MPTP groups. (**F**) SNr TH immunoreactivity data. Data are shown as mean ± S.E.M. (n = 10). ***P < 0.001. Bar = 50 μm.

A repeated measures two-way ANOVA revealed that treatment with an antibiotic cocktail for 14 days reduced mouse body weights (antibiotic: F_1,36_ = 20.549, P < 0.001; MPTP: F_1,36_ = 0.005, P = 0.994; interaction (antibiotic × MPTP): F_1,36_ = 0.045, P = 0.833; Figure 1B). On day 22, antibiotic + saline group body weights, but not antibiotic + MPTP group weights, remained lower than those of mice that did not receive antibiotics (Figure 1B). Thus, antibiotic + MPTP group body weights recovered gradually after treatment, while antibiotic + saline group weights did not.

### Antibiotic treatment protected against MPTP-induced neurotoxicity in the mouse brain

DAT immunohistochemistry revealed that MPTP reduced DAT levels in the striatum of the water-treated group, but not the antibiotic-treated group (Figure 1C). A two-way ANOVA revealed significant differences in DAT immunoreactivity among the four groups (antibiotic: F_1,36_ = 11.30, P = 0.002; MPTP: F_1,36_ = 20.46, P < 0.001; interaction (antibiotic × MPTP): F_1,36_ = 15.32, P < 0.001; Figure 1D). TH immunohistochemistry revealed that MPTP reduced TH immunoreactivity in the SNr of the water-treated group, but not the antibiotic-treated group (Figure 1E). A two-way ANOVA revealed significant differences in TH immunoreactivity among the four groups (antibiotic: F_1,36_ = 11.48, P = 0.002; MPTP: F_1,36_ = 10.19, P = 0.003; interaction (antibiotic × MPTP): F_1,36_ = 11.75, P = 0.002; Figure 1F). Collectively, these results indicate that treatment with an antibiotic cocktail for 14 days protected against MPTP-induced dopaminergic neurotoxicity in the striatum and SNr.

### Gut microbiota composition

**Figure 2:**
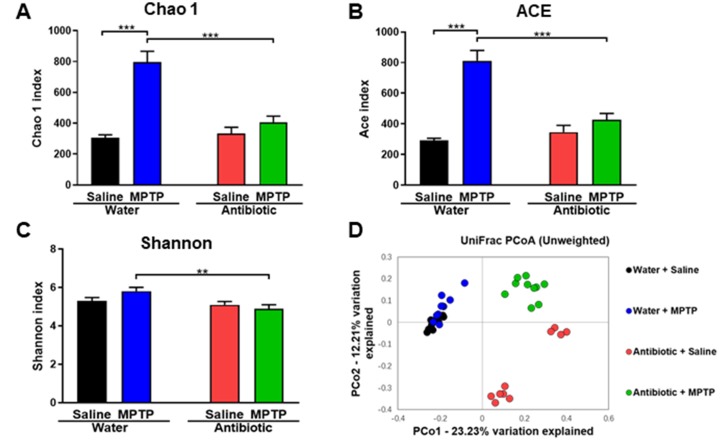
**α-diversity and β-diversity in gut microbiota.** Diversity index values for the four groups. (**A**) *Chao 1* index (two-way ANOVA, antibiotic: F_1,36_ = 15.928, P < 0.001; MPTP: F_1,36_ = 37.541, P < 0.001; interaction (antibiotic × MPTP): F_1,36_ = 20.587, P < 0.001). (**B**) *ACE* index (two-way ANOVA, antibiotic: F_1,36_ = 12.968, P < 0.001; MPTP: F_1,36_ = 43.032, P < 0.001; interaction (antibiotic × MPTP): F_1,36_ = 22.827, P < 0.001). (**C**) *Shannon* index (two-way ANOVA, antibiotic: F_1,36_ = 8.942, P = 0.005; MPTP: F_1,36_ = 0.593, P = 0.446; interaction (antibiotic × MPTP): F_1,36_ = 3.427, P = 0.072). (**D**) PCoA analysis of gut bacteria data (Bray–Curtis dissimilarity).

Next, we investigated the composition of the gut microbiota, which can be altered by antibiotic cocktail treatment [33–35], in the four experimental groups. α-diversity is defined as the richness of gut microbiota and can be measured using different indices. Two-way ANOVAs revealed a significant difference in the Chao1 (antibiotic: F_1,36_ = 15.928, P < 0.001; MPTP: F_1,36_ = 37.541, P < 0.001; interaction (antibiotic × MPTP): F_1,36_ = 20.587, P < 0.001; Figure 2A) and Ace (antibiotic: F_1,36_ = 12.968, P < 0.001; MPTP: F_1,36_ = 43.032, P < 0.001; interaction (antibiotic × MPTP): F_1,36_ = 22.827, P < 0.001; Figure 2B) indices among the four groups. Specifically, Chao 1 and Ace indices were higher in the water + MPTP group than in both the water + saline and antibiotic + MPTP groups (P < 0.001). Interestingly, antibiotic cocktail treatment attenuated the MPTP-induced increase in the Chao 1 and Ace indices. A two-way ANOVA also revealed significant differences in the Shannon index among the four groups (antibiotic: F_1,36_ = 8.942, P = 0.005; MPTP: F_1,36_ = 0.593, P = 0.446; interaction (antibiotic × MPTP): F_1,36_ = 3.427, P = 0.072; Figure 2C). The Shannon index in the antibiotic + MPTP group was lower than that of the water + MPTP group. In an unweighted UniFrac PCoA dot map, dots representing the antibiotic-treated groups were far away from dots representing the water-treated groups (Figure 2D). Interestingly, dots representing the antibiotic + MPTP group were isolated from dots representing the other three groups (Figure 2D).

**Figure 3:**
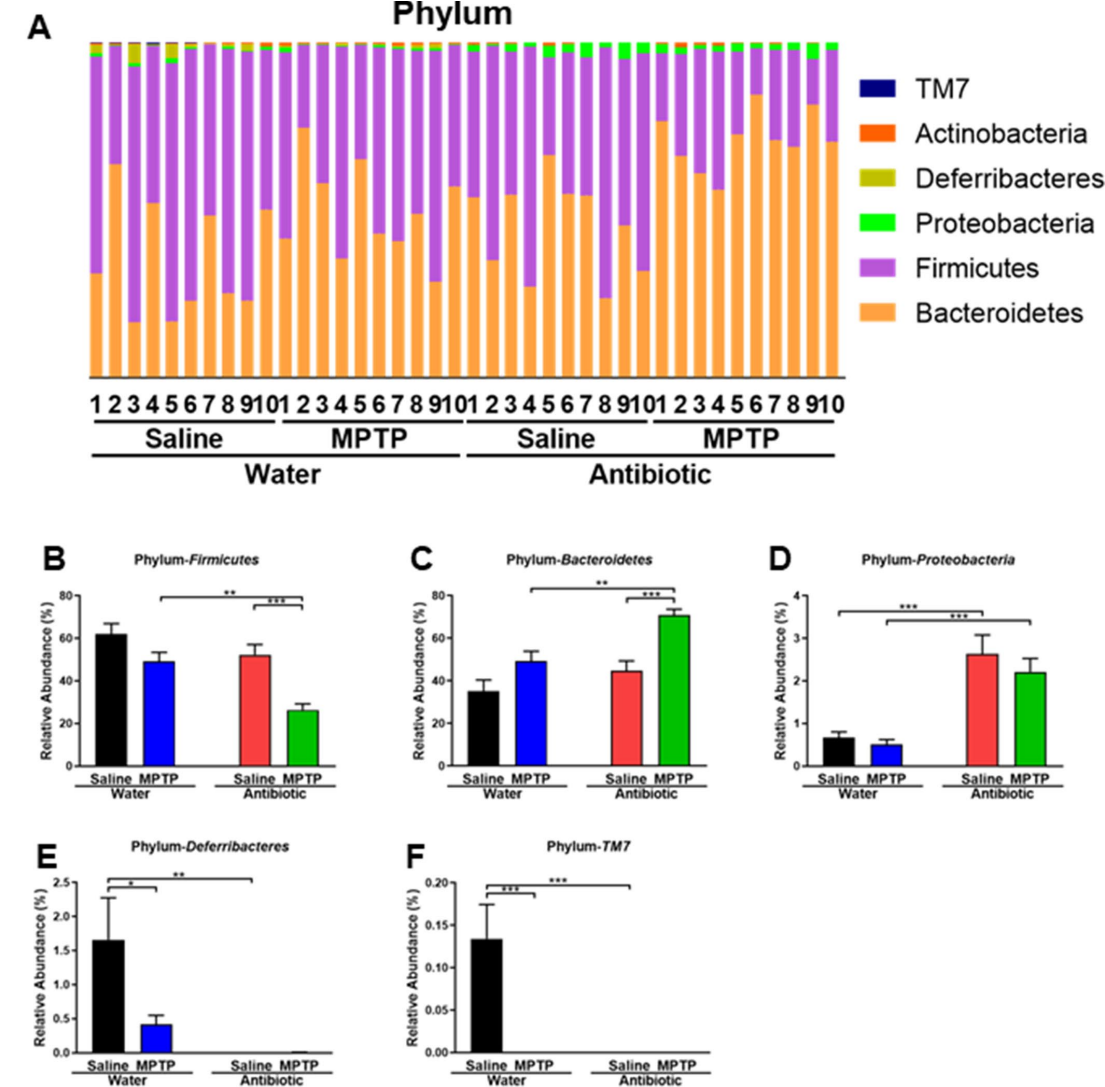
**Altered gut bacteria composition at the phylum level.** (**A**) Relative abundance at the phylum level. (**B**) *Bacteroidetes*. (**C**) *Firmicutes*. (**D**) *Proteobacteria*. (**E**) *Deferribacteres*. (**F**) *TM7*. Data are shown as mean ± S.E.M. (n = 10). **P < 0.01, ***P < 0.001. See the Supplementary Table 1 for detailed statistical analysis.

At the phylum level, *Firmicutes* were the most abundant phylum in the water + saline group microbiota (Figure 3A and 3B). The abundance of *Firmicutes* was lower in the antibiotic + MPTP group than in the water + MPTP and antibiotic + saline groups (P < 0.001, Figure 3B). In contrast, the most dominant phylum in the antibiotic + MPTP group, *Bacteroidetes*, was less abundant in the water + MPTP and antibiotic + saline groups (P < 0.001, Figure 3C). *Proteobacteria* levels were higher after treatment with the antibiotic cocktail compared to the two water-treated groups (Figure 3D), while *Deferribacteres* and *TM7* levels decreased after treatment with the antibiotic cocktail or with MPTP (Figure 3E, 3F).

**Figure 4:**
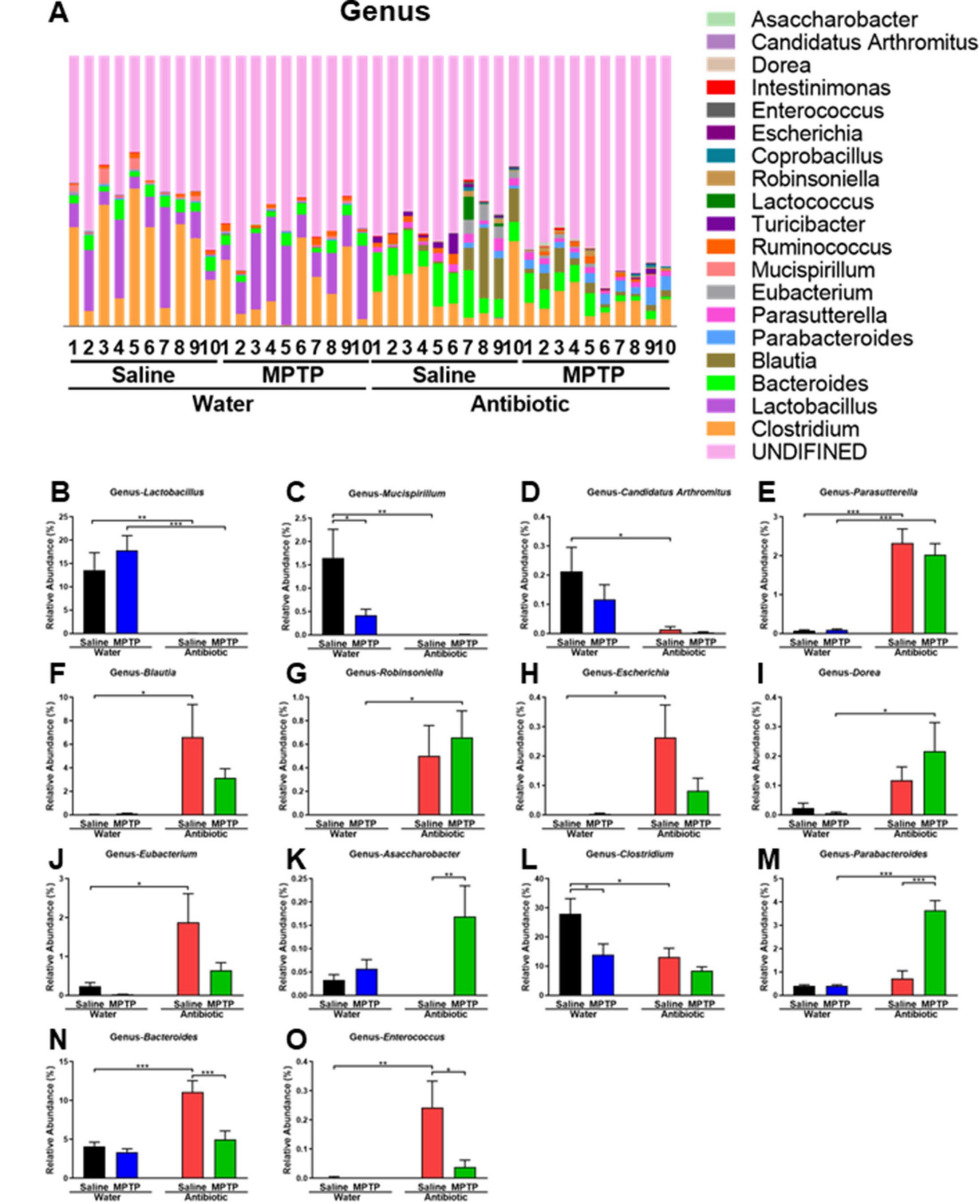
**Altered gut bacteria composition at the genus level.** (**A**) Relative abundance at the genus level. (**B**) *Lactobacillus*. (**C**) *Mucispirillum*. (**D**) *Candidatus Arthromitus*. (**E**) *Parasutterella*. (**F**) *Blautia*. (**G**) *Robinsoniella*. (**H**) *Escherichia*. (**I**) *Dorea*. (**J**) *Eubacterium*. (**K**) *Asaccharobacter*. (**L**) *Clostridium*. (**M**) *Parabacteroides*. (**N**) *Bacteroides*. (**O**) *Enterococcus*. Data are shown as mean ± S.E.M. (n = 10). **P < 0.01, ***P < 0.001. See the Supplementary Table 2 for detailed statistical analysis.

Antibiotic and MPTP treatment also altered the composition of fecal microbiota at the genus level (Figure 4A). *Lactobacillus,* *Mucispirillum*, and *Candidatus Arthromitus* levels decreased after treatment with the antibiotic cocktail (Figure 4B–4D). In contrast, *Parasutterella, Blautia, Robinsoniella, Escherichia, Dorea*, and *Eubacterium* levels increased after treatment with antibiotic cocktail (Figure 4E–4J). Interestingly, *Asaccharobacter* levels increased in the antibiotic + MPTP group compared to the other three groups (Figure 4K). *Clostridium,* which was the most dominant genus in control mice, decreased after MPTP injections and antibiotic cocktail treatment (Figure 4L). Furthermore, the antibiotic + MPTP group had a higher abundance of *Parabacteroides* than the other three groups (Figure 4M). Finally, MPTP treatment attenuated the antibiotic-induced increase in the abundance of *Bacteroides* and *Enterococcus* (Figure 4N, 4O).

**Figure 5:**
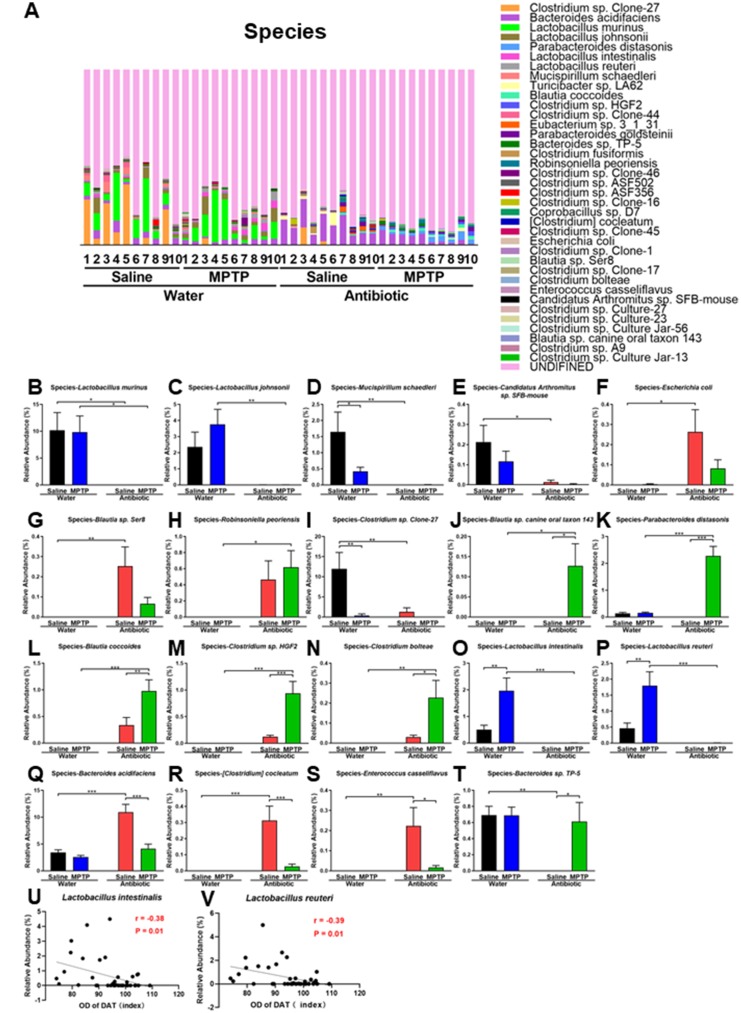
**Altered gut bacteria composition at the species level.** (**A**) Relative abundance at the species level. (**B**) *Lactobacillus murinus.* (**C**) *Lactobacillus johnsonii.* (**D**) *Mucispirillum schaedleri.* (**E**) *Candidatus Arthromitus sp. SFB-mouse*. (**F**) *Escherichia coli*. (**G**) *Blautia sp. Ser8.* (**H**) *Robinsoniella peoriensis.* (**I**) *Clostridium sp. Clone-27.* (**J**) *Blautia sp. canine oral taxon 143.* (**K**) *Parabacteroides distasonis*. (**L**) *Blautia coccoides.* (**M**) *Clostridium sp. HGF2.* (**N**) *Clostridium bolteae*. (**O**) *Lactobacillus intestinalis*. (**P**) *Lactobacillus reuteri*. (**Q**) *Bacteroides acidifaciens.* (**R**) *[Clostridium] cocleatum.* (**S**) *Enterococcus casseliflavus*. (**T**) *Bacteroides sp. TP-5.* (**U**) Negative correlation (r = -0.38, P = 0.01) between *Lactobacillus intestinalis* and DAT immunoreactivity*.* (**V**) Negative correlation (r = - 0.39, P = 0.01) between *Lactobacillus reuteri* and DAT immunoreactivity. Data are shown as mean ± S.E.M. (n = 10). *P < 0.05, **P < 0.01, ***P < 0.001. See the Supplementary Table 3 for detailed statistical analysis.

Gut microbiota composition at the species level in the four experimental groups is shown in Figure 5A. *Lactobacillus murinus, Lactobacillus johnsonii, Mucispirillum schaedleri*, and *Candidatus Arthromitus sp. SFB-mouse* decreased after antibiotic cocktail treatment (Figure 5B–5E). In contrast, *Escherichia coli, Blautia sp. Ser8,* and *Robinsoniella peoriensis* increased after antibiotic treatment (Figure 5F–5H). *Clostridium sp. Clone-27*, the most abundant species in control water + saline group mice, decreased in all other groups (Figure 5I). On the other hand, *Blautia sp. canine oral taxon 143, Parabacteroides distasonis, Blautia coccoides, Clostridium sp. HGF2*, and *Clostridium bolteae* increased in the antibiotic + MPTP group (Figure 5J–5N). In addition, *Lactobacillus intestinalis* and *Lactobacillus reuteri*, which increased in the water + MPTP group compared to the water + saline group, were markedly reduced after antibiotic treatment (Figure 5O and 5P). Interestingly, DAT immunoreactivity was negatively correlated with levels of *Lactobacillus intestinalis* (r = -0.38, P = 0.01) and *Lactobacillus reuteri* (r = -0.39, P = 0.01; Figure 5U and 5V). The antibiotic-induced increase in the abundance of *Bacteroides acidifaciens, [Clostridium] cocleatum*, and *Enterococcus casseliflavus* was largely reversed after MPTP administration (Figure 5Q–5S). In addition, MPTP restored *Bacteroides sp. TP-5* to control levels after it had been reduced by antibiotic treatment (Figure 5T).

## DISCUSSION

In this study, we examined the effects of treatment with an antibiotic cocktail on gut microbiota in a mouse model of PD. We found that MPTP markedly reduced DAT immunoreactivity in the striatum and TH immunoreactivity in the SNr of the water-treated group, but not the antibiotic-treated group. Second, antibiotic cocktail treatment caused substantial alterations in host gut microbiota composition compared to the water-treated group. In an unweighted UniFrac PCoA, dots representing the two antibiotic-treated groups were located far away from dots representing the two water-treated groups. Interestingly, dots representing the antibiotic + MPTP group were also far from the dots of the other three groups. At the phylum level, *Proteobacteria* was markedly increased, while *Deferribacteres,* and *TM7* were markedly decreased, in the gut of antibiotic-treated mice. Antibiotic treatment was also associated with substantial microbiome alterations at the genus and species levels. Overall, 14 days of treatment with an antibiotic cocktail caused significant changes in the diversity and composition of the host gut microbiota, which is consistent with previous reports [33–35]. Taken together, these results suggest that antibiotic–induced microbiome depletion might protect against MPTP-induced dopaminergic neurotoxicity in the mouse brain via the brain–gut microbiota axis.

In another recent study, we reported that 14 days of antibiotic treatment increases levels of bacteria from the phylum *Proteobacteria* and decreases levels of the major bacterial phyla *Bacteroidetes* and *Firmicutes* in the mouse gut microbiota (Wang *et* *al*., submitted); similar results have also been observed in other studies [26, 31, 32]. Here, we found that the relative abundance of *Proteobacteria* increased in the antibiotic-treated groups compared to the water-treated groups. In contrast, the relative abundance of *Firmicutes* and *Bacteroidetes* was similar in the antibiotic + saline and water + saline groups, indicating that spontaneous recovery of these bacteria occurred. The mechanisms underlying the increased relative abundance of *Proteobacteria* after antibiotic cocktail treatment are currently unknown. Interestingly, MPTP significantly altered the relative abundance of *Firmicutes* and *Bacteroidetes* in the antibiotic-treated group, but not in the water-treated group. Thus, MPTP might further alter gut microbiome composition after antibiotic-induced microbiome depletion.

MPTP specifically increased levels of the following bacterial species in the guts of antibiotic-treated mice: *Blautia sp. Canine oral taxon 143, Parabacteroides distasonis, Blautia coccoides, Clostridium sp. HGF2, Clostridium bolteae,* and *Bacteroides sp. TP-5*. A recent study demonstrated that *Parabacteroides distasonis* alleviated obesity and metabolic dysfunction via production of succinate and secondary bile acids [38]. In addition, *Parabacteroides distasonis* reduced the severity of intestinal inflammation in murine models of acute and chronic colitis induced by dextran sulphate sodium, suggesting that *Parabacteroides distasonis* may be useful for treating inflammatory bowel diseases [39]. *Blautia coccoides* produce hydrogen [13], which might have beneficial effects in the MPTP mouse model [40]. Interestingly, *Clostridium sp. HGF2* plays an important role in the metabolism of mannitol [41], which could attenuate behavioral abnormalities and aggregations of α-synuclein in the rodent brain [42, 43]. Among the bacteria increased by MPTP, *Bacteroides sp. TP-5* is particularly noteworthy due to its ability to modulate immune system function [44]. Furthermore, a recent study demonstrated that fecal microbiota transplantation protected against MPTP-induced neurotoxicity by suppressing neuroinflammation [45]. The effects of supplementation with *Bacteroides sp. TP-5* on dopaminergic neurotoxicity in mouse MPTP model should be investigated further.

MPTP treatment also decreased levels of the *Bacteroides acidifaciens, [Clostridium] cocleatum,* and *Enterococcus casseliflavus* bacterial species. *Bacteroides acidifaciens* are important for promoting IgA production in the large intestine [46]. Interestingly, *Bacteroides acidifaciens* levels were increased in the feces of *Atg7*^ΔCD11c^ mice with a lean phenotype compared to those of control *Atg7*^f/f^ mice, and wild-type C57BL/6 mice fed with a diet including *Bacteroides acidifaciens* gained less weight and fat mass than mice fed control food [47]. Those results suggest that *Bacteroides acidifaciens* might be a potential treatment for metabolic diseases such as obesity [47]. The functional roles of *[Clostridium] cocleatum* and *Enterococcus casseliflavus* are unclear, and the mechanisms underlying the recovery of the three bacterial species that increased in the gut microbiome of antibiotic-treated mice after MPTP administration in this study are currently unknown. Regardless, it is likely that interactions between the brain–gut axis and these microbiomes play a role in MPTP-induced neurotoxicity, and the relationship between neuroprotection, the immune system, and the brain–gut axis warrants further investigation.

In this study, we found that DAT immunoreactivity was negatively correlated with *Lactobacillus intestinalis* and *Lactobacillus reuteri* levels despite the marked decrease observed in these species after antibiotic treatment*.* Both of these bacteria produce lactic acid, which was more abundant in the striatum of MPTP-treated mice [48]. Furthermore, treatment with *Lactobacillus reuteri* selectively rescues social deficits in genetic, environmental, and idiopathic autism spectrum disorder (ASD) models, suggesting that this species may be a promising non-invasive microbial-based therapy for ASD-related social dysfunctions [49]. Additionally, short-chain fatty acids promote proliferation of *Lactobacillus reuteri* [50]; this effect should be investigated further. It is possible that *Lactobacillus intestinalis*, *Lactobacillus reuteri*, and lactic acid might affect the dopaminergic neurotoxicity of MPTP in the brain. Furthermore, antibiotic-induced microbiome depletion might enhance or counteract MPTP-induced dopaminergic neurotoxicity in mouse brain, although the specific microbes that might be involved in these effects were not identified in this study. Additional studies are needed to confirm the relationship between MPTP-induced dopaminergic neurotoxicity and the gut microbiome.

Accumulating evidence suggests that abnormal gut microbiota composition might affect neuroprotection [8, 51, 52]. Choi *et* *al*. [53] identified dramatic and widespread increases in levels of *Enterobacteriaceae*, and particularly of *Proteus mirabilis*, in the mouse MPTP model. Administration of *Proteus mirabilis* isolated from MPTP-treated mice produced motor deficits, dopaminergic neuronal damage, and inflammation in the striatum and SNr, suggesting that *Proteus mirabilis* promotes PD pathology in the brain. Furthermore, Srivastav *et* *al*. [54] reported neuroprotective effects of a probiotic cocktail containing *Lactobacillus rhamnosus GG*, *Bifidobacterium animalis lactis*, and *Lactobacillus acidophilus* in MPTP-treated mice. Together, these results indicate that altered gut microbiota composition likely plays a role in dopaminergic neurotoxicity related to PD.

In conclusion, the present study suggests that antibiotic-induced microbiome depletion might protect against MPTP-induced dopaminergic neurotoxicity in the mouse brain, and that MPTP might improve the diversity and composition of gut microbiota in antibiotic-treated mice. These results indicate that the brain–gut axis plays a key role in the pathology of PD.

## MATERIALS AND METHODS

### Animals

Male adult C57BL/6 mice (8 weeks old) weighting 20-25 g were purchased from SLC (Inc., Hamamatsu, Japan). Animals were housed under controlled temperatures and 12-hour light/dark cycles (lights on between 07:00–19:00) with ad libitum food (CE-2; CLEA Japan, Inc., Tokyo, Japan) and water. All experiments were carried out according to the Guide for Animal Experimentation of Chiba University. The experimental protocol was approved by the Chiba University Institutional Animal Care and Use Committee.

### Preparation of antibiotics and MPTP

As described in previous reports [33–35], broad-spectrum antibiotics (ampicillin 1 g/L, neomycin sulfate 1 g/L, metronidazole 1 g/L, Sigma-Aldrich Co., Ltd, St Louis, MO, USA) were dissolved in drinking water. This antibiotic cocktail, which was prepared fresh every other day, was administered to adult C57BL/6 mice for 14 continuous days. 1-Methyl-4-phenyl-1,2,3,6-tetrahydropyridine (MPTP: Tokyo Chemical Industry CO., Ltd., Tokyo, Japan) was dissolved in saline. Other compounds were purchased commercially.

### Schedule of treatment and collection of fecal and brain samples

The procedure for establishing MPTP-induced neurotoxicity was performed as previously reported [55, 56]. Forty mice (8 weeks old) were divided among the following four groups: water + saline; water + MPTP; antibiotic cocktail + saline; antibiotic cocktail + MPTP. Mice were given drinking water with or without the antibiotic cocktail from day 1 to day 14 (Figure 1A). All mice were given water without antibiotics from day 15 to day 22. On day 15, mice received intraperitoneal injections of MPTP (10 mg/kg x 3, 2-hr interval) or saline (5 mL/kg x 3, 2-hr interval; Figure 1A). One week after MPTP or saline injection, fresh fecal samples were collected and stored at -80°C until use. Subsequently, the mice were anesthetized with 5% isoflurane and sodium pentobarbital (50 mg/kg) for brain collection. Mice were perfused transcardially with 10 mL of isotonic saline followed by 30 mL of ice-cold 4% paraformaldehyde in 0.1 M phosphate buffer (pH 7.4). Brains were removed, post-fixed overnight at 4°C, and then used for immunohistochemical stating of dopamine transporter (DAT) and tyrosine hydroxylase (TH).

### DAT and TH Immunohistochemistry

Immunohistochemical staining of DAT and TH was performed as reported previously [55, 56]. Consecutive 50 μm-thick coronal brain sections (bregma 0.86−1.54 mm and -2.92–3.88 mm) were cut in ice-cold 10 mM phosphate buffered saline (pH 7.5) using a vibrating blade microtome (VT1000s, Leica Microsystems AG, Wetzlar, Germany). Free-floating sections were treated with 0.3% H_2_O_2_ in 50 mM Tris-HCL saline (TBS) for 30 min and blocked in 0.2% Triton X-100 TBS (TBST) with 1.5% normal serum for 1 hour at room temperature. Samples were then incubated for 36 hours at 4°C with rat anti-DAT antibody (1:10,000, Merck Millipore, Burlington, MA, USA) or rabbit anti-TH antibody (1:500, Sigma-Aldrich, St Louis, MO, USA). The sections were then washed three times in TBS and processed according to the avidin-biotin-peroxidase method (Vectastain Elite ABC, Vector Laboratories, Inc., Burlingame, CA, USA). Sections were then incubated with 0.15 mg/mL diaminobenzidine and 0.01% H_2_O_2_ for 5 minutes; the staining solution for DAT only also contained 0.06% NiCl_2_. The sections were then mounted on gelatinized slides, dehydrated, cleared, and coverslipped with Permount^®^ (Fisher Scientific, Fair Lawn, NJ, USA). Images were taken and DAT and TH immunoreactivity staining intensity in the anterior region (0.25 mm^2^) of the striatum as well as the number of TH-positive cells in SNr region (0.36 mm^2^) were analyzed using a Keyence BZ-9000 Generation microscope (Keyence Co., Ltd, Osaka, Japan). Eight data points (four brain slides) from each mouse were used for the quantitative analyses of DAT and TH immunoreactivity.

### 16S rRNA analysis

DNA was extracted from fecal samples and 16S rRNA analyses were performed as previously described [57] by MyMetagenome Co., Ltd. (Tokyo, Japan). Briefly, PCR was performed using 27Fmod 5′-AGRGTTTGATYM TGGCTCAG-3′ and 338R 5′-TGCTGCCTCCCGTAGG AGT-3′ primers to amplify the V1–V2 region of the bacterial 16S rRNA gene. The amplified DNA (~330bp) was purified using AMPure XP (Beckman Coulter) and quantified using a Quant-iT Picogreen dsDNA assay kit (Invitrogen) and a TBS-380 Mini-Fluorometer (Turner Biosystems). The 16S amplicons were then sequenced using a MiSeq according to the Illumina protocol. The paired-end reads were merged using the fastq-join program based on overlapping sequences. Reads with an average quality value of <25 and inexact matches to both universal primers were filtered out. Filter-passed reads were analyzed further after trimming off both primer sequences. For each sample, 3,000 high-quality filter-passed reads were rearranged in descending order according to quality value and then clustered into operational taxonomic units (OTUs) with a 97% pairwise-identity cutoff using the UCLUST program version 5.2.32 (https://www.drive5.com). Taxonomic assignments of OTUs were performed based on similarity searches against the Ribosomal Database Project and the National Center for Biotechnology Information genome database using the GLSEARCH program [58].

### Statistical analysis

Animal experiment data are presented as the mean ± standard error of the mean (S.E.M.). Statistical analyses were performed using SPSS Statistics 20 (SPSS, Tokyo, Japan). Body weight data were analyzed using repeated two-way analysis of variance (ANOVA) followed by *post-hoc* Tukey’s multiple comparison tests. DAT and TH immunohistochemistry and 16S rDNA data were analyzed using two-way ANOVAs followed by *post-hoc* Tukey’s multiple comparison tests. *P* values of less than 0.05 were considered statistically significant.

## Supplementary Material

Supplementary Tables
