# Supplementary material for: Antibiotic-induced microbiome depletion protects against MPTP-induced dopaminergic neurotoxicity in the brain
Source: Aging (Albany NY). 2019 Sep 3;11(17):6915–29. doi: 10.18632/aging.102221 (PMC6756889; doi:10.18632/aging.102221)
Supplement: Supplementary Tables [file aging-11-102221-s001.pdf]

SUPPLEMENTARY TABLES

Supplementary Table 1. Statistical analysis data of gut microbiota data at phylum.

Graph	Factor effect (Antibiotic)	Factor effect (MPTP)	Interaction effect (Antibiotic × MPTP)
Bacteroidetes	$F_{(1,36)} = 12.695$, $P = 0.001$	$F_{(1,36)} = 21.180$, $P < 0.001$	$F_{(1,36)} = 1.806$, $P = 0.187$
Firmicutes	$F_{(1,36)} = 14.284$, $P = 0.001$	$F_{(1,36)} = 20.353$, $P < 0.001$	$F_{(1,36)} = 2.272$, $P = 0.140$
Proteobacteria	$F_{(1,36)} = 40.495$, $P < 0.001$	$F_{(1,36)} = 1.034$, $P = 0.316$	$F_{(1,36)} = 0.190$, $P = 0.665$
Deferribacteres	$F_{(1,36)} = 10.911$, $P = 0.002$	$F_{(1,36)} = 3.872$, $P = 0.057$	$F_{(1,36)} = 3.948$, $P = 0.055$
TM7	$F_{(1,36)} = 10.916$, $P = 0.002$	$F_{(1,36)} = 10.916$, $P = 0.002$	$F_{(1,36)} = 10.916$, $P = 0.002$

Supplementary Table 2. Statistical analysis data of gut microbiota data at genus.

Graph	Factor effect (Antibiotic)	Factor effect (MPTP)	Interaction effect (Antibiotic × MPTP)
lactobacillus	$F_{(1,36)} = 40.826$, $P < 0.001$	$F_{(1,36)} = 0.743$, $P = 0.394$	$F_{(1,36)} = 0.744$, $P = 0.394$
Mucispirillum	$F_{(1,36)} = 10.835$, $P = 0.002$	$F_{(1,36)} = 3.818$, $P = 0.059$	$F_{(1,36)} = 3.893$, $P = 0.056$
Candidatus Arthromitus	$F_{(1,36)} = 10.302$, $P = 0.003$	$F_{(1,36)} = 1.204$, $P = 0.280$	$F_{(1,36)} = 0.796$, $P = 0.378$
Parasutterella	$F_{(1,36)} = 81.500$, $P < 0.001$	$F_{(1,36)} = 0.344$, $P = 0.561$	$F_{(1,36)} = 0.495$, $P = 0.486$
Blautia	$F_{(1,36)} = 11.239$, $P = 0.002$	$F_{(1,36)} = 1.430$, $P = 0.240$	$F_{(1,36)} = 1.542$, $P = 0.222$
Robinsoniella	$F_{(1,36)} = 11.589$, $P = 0.002$	$F_{(1,36)} = 0.211$, $P = 0.649$	$F_{(1,36)} = 0.211$, $P = 0.649$
Escherichia	$F_{(1,36)} = 8.289$, $P = 0.007$	$F_{(1,36)} = 2.245$, $P = 0.143$	$F_{(1,36)} = 2.399$, $P = 0.130$
Dorea	$F_{(1,36)} = 7.766$, $P = 0.008$	$F_{(1,36)} = 0.548$, $P = 0.464$	$F_{(1,36)} = 1.104$, $P = 0.300$
Eubacterium	$F_{(1,36)} = 8.529$, $P = 0.006$	$F_{(1,36)} = 3.508$, $P = 0.069$	$F_{(1,36)} = 1.747$, $P = 0.195$
Asaccharobacter	$F_{(1,36)} = 1.292$, $P = 0.263$	$F_{(1,36)} = 7.712$, $P = 0.009$	$F_{(1,36)} = 4.353$, $P = 0.044$
Clostridium	$F_{(1,36)} = 7.982$, $P = 0.008$	$F_{(1,36)} = 6.757$, $P = 0.013$	$F_{(1,36)} = 1.664$, $P = 0.205$
Parabacteroides	$F_{(1,36)} = 43.663$, $P < 0.001$	$F_{(1,36)} = 29.742$, $P < 0.001$	$F_{(1,36)} = 29.742$, $P < 0.001$
Bacteroides	$F_{(1,36)} = 19.018$, $P < 0.001$	$F_{(1,36)} = 12.176$, $P = 0.001$	$F_{(1,36)} = 7.447$, $P = 0.010$
Enterococcus	$F_{(1,36)} = 8.910$, $P = 0.005$	$F_{(1,36)} = 4.904$, $P = 0.033$	$F_{(1,36)} = 4.624$, $P = 0.038$

Supplementary Table 3. Statistical analysis data of gut microbiota data at species.

Graph	Factor effect (Antibiotic)	Factor effect (MPTP)	Interaction effect (Antibiotic × MPTP)
Lactobacillus murinus	$F_{(1,36)}$ = 19.973, P < 0.001	$F_{(1,36)}$ = 0.007, P = 0.935	$F_{(1,36)}$ = 0.006, P = 0.936
Lactobacillus johnsonii	$F_{(1,36)}$ = 21.666, P < 0.001	$F_{(1,36)}$ = 1.126, P = 0.296	$F_{(1,36)}$ = 1.126, P = 0.296
Mucispirillum schaedleri	$F_{(1,36)}$ = 10.803, P = 0.002	$F_{(1,36)}$ = 3.836, P = 0.058	$F_{(1,36)}$ = 3.912, P = 0.056
Candidatus Arthromitus sp. SFB-mouse	$F_{(1,36)}$ = 10.302, P = 0.003	$F_{(1,36)}$ = 1.204, P = 0.280	$F_{(1,36)}$ = 0.796, P = 0.378
Escherichia coli	$F_{(1,36)}$ = 8.289, P = 0.007	$F_{(1,36)}$ = 2.245, P = 0.143	$F_{(1,36)}$ = 2.399, P = 0.130
Blautia sp. Ser8	$F_{(1,36)}$ = 10.059, P = 0.003	$F_{(1,36)}$ = 3.457, P = 0.071	$F_{(1,36)}$ = 3.457, P = 0.071
Robinsoniella peoriensis	$F_{(1,36)}$ = 11.921, P = 0.001	$F_{(1,36)}$ = 0.239, P = 0.628	$F_{(1,36)}$ = 0.239, P = 0.628
Clostridium sp. Clone-27	$F_{(1,36)}$ = 6.957, P = 0.012	$F_{(1,36)}$ = 9.366, P = 0.004	$F_{(1,36)}$ = 6.036, P = 0.019
Blautia sp. canine oral taxon 143	$F_{(1,36)}$ = 5.309, P = 0.027	$F_{(1,36)}$ = 5.309, P = 0.027	$F_{(1,36)}$ = 5.309, P = 0.027
Parabacteroides distasonis	$F_{(1,36)}$ = 30.573, P < 0.001	$F_{(1,36)}$ = 40.561, P < 0.001	$F_{(1,36)}$ = 39.576, P < 0.001
Blautia coccoides	$F_{(1,36)}$ = 26.794, P < 0.001	$F_{(1,36)}$ = 6.406, P = 0.016	$F_{(1,36)}$ = 6.406, P = 0.016
Clostridium sp. HGF2	$F_{(1,36)}$ = 21.898, P < 0.001	$F_{(1,36)}$ = 12.850, P = 0.001	$F_{(1,36)}$ = 12.850, P = 0.001
Clostridium bolteae	$F_{(1,36)}$ = 8.670, P = 0.006	$F_{(1,36)}$ = 5.094, P = 0.030	$F_{(1,36)}$ = 5.094, P = 0.030
Lactobacillus intestinalis	$F_{(1,36)}$ = 23.328, P < 0.001	$F_{(1,36)}$ = 8.172, P = 0.007	$F_{(1,36)}$ = 8.105, P = 0.007
Lactobacillus reuteri	$F_{(1,36)}$ = 23.676, P < 0.001	$F_{(1,36)}$ = 8.288, P = 0.007	$F_{(1,36)}$ = 8.214, P = 0.007
Bacteroides acidifaciens	$F_{(1,36)}$ = 26.102, P < 0.001	$F_{(1,36)}$ = 18.528, P < 0.001	$F_{(1,36)}$ = 11.141, P = 0.002
[Clostridium] cocleatum	$F_{(1,36)}$ = 14.097, P = 0.001	$F_{(1,36)}$ = 9.975, P = 0.003	$F_{(1,36)}$ = 9.975, P = 0.003
Enterococcus casseliflavus	$F_{(1,36)}$ = 6.743, P = 0.014	$F_{(1,36)}$ = 5.058, P = 0.031	$F_{(1,36)}$ = 5.058, P = 0.031
Bacteroides sp. TP-5	$F_{(1,36)}$ = 7.426, P = 0.010	$F_{(1,36)}$ = 4.685, P = 0.037	$F_{(1,36)}$ = 4.747, P = 0.036
